# Virtual Surgical Planning: Modeling from the Present to the Future

**DOI:** 10.3390/jcm10235655

**Published:** 2021-11-30

**Authors:** G. Dave Singh, Manarshhjot Singh

**Affiliations:** 1 Virtual Craniofacial Laboratory, Stanford University, Stanford, CA 94301, USA; 2 UMR 9189 CRIStAL, Université de Lille, F-59000 Lille, France; manarshhjot.singh@gmail.com

**Keywords:** virtual surgery planning, landmark data, predictive modeling

## Abstract

Virtual surgery planning is a non-invasive procedure, which uses digital clinical data for diagnostic, procedure selection and treatment planning purposes, including the forecast of potential outcomes. The technique begins with 3D data acquisition, using various methods, which may or may not utilize ionizing radiation, such as 3D stereophotogrammetry, 3D cone-beam CT scans, etc. Regardless of the imaging technique selected, landmark selection, whether it is manual or automated, is the key to transforming clinical data into objects that can be interrogated in virtual space. As a prerequisite, the data require alignment and correspondence such that pre- and post-operative configurations can be compared in real and statistical shape space. In addition, these data permit predictive modeling, using either model-based, data-based or hybrid modeling. These approaches provide perspectives for the development of customized surgical procedures and medical devices with accuracy, precision and intelligence. Therefore, this review briefly summarizes the current state of virtual surgery planning.

## 1. Introduction

Virtual surgical planning (VSP) addresses operative procedures using digital data. The primary objective of VSP is to improve the clinical workflow, but it may also help in selecting/customizing a procedure for a specific patient. These objectives can be met in a variety of ways since VSP can be used for pre-operative planning, decreasing surgery time and visualization of potential post-operative outcomes. A common factor that allows for the above functionalities is the ability of automating VSP without compromising accuracy. VSP can be combined with other techniques such as 3D printing to create patient specific surgery tools, implants, and virtual reality with or without haptics for training purposes. To achieve these tasks, VSP requires robust techniques to interrogate pre-treatment data to predict post-treatment configurations. Unlike 2D cephalometric techniques, the fundamentals of VSP methods rely on industry standard imaging protocols (such as DICOM) to: permit data integration from different sources; select the global reference frame and simulate surgical movements prior to designing and fabricating surgical splints for craniofacial, orthognathic and maxillofacial surgery inter alia.

## 2. Virtual Surgery Planning Procedure

**Figure 1:**
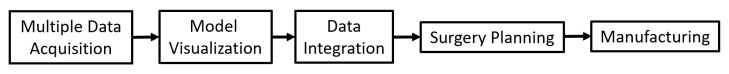
Schematic of virtual surgery planning procedure.

Although the overall procedure of VSP is similar in most cases, it can be affected if patient-specific tooling needs to be designed. Thus, various steps are necessary for the proper implementation of VSP prior to any type of surgical procedure. The overall schematic for steps in VSP is shown in Figure 1.

### 2.1. Data Acquisition

Data acquisition is the critical first stage of VSP, and standardization of image capture is strongly suggested to provide consistent measures. The data include the shape and size information of the tissues, but this can be influenced by patient positioning during data capture. Information on the relative location and orientation of the surgical site might be required in order to compare it to other tissues. In addition, since both 2D and 3D data can be used for VSP, data can be acquired from one or more sources and combined or integrated.

1. Radiographs: Traditional 2D radiographs are an old but common method of visualizing the bony tissues to enable their comparison with adjacent soft tissues. Radiographs provide the hard and soft tissue shape and size information in 2D, which can be used in conjunction with 2D photographs to visualize the planned surgical outcome. However, providing only 2D information about shape and size is a significant limitation since VSP requires specific movements, and alignment from planar information is a difficult task that requires skill and experience. Moreover, cephalometric 2D data do not exist in 3D space and are considered by some to be inappropriate for clinical diagnosis and treatment planning [1,2].
2. Photographs: 2D photography is the easiest, least invasive and a low-cost source of collecting facial information. For cranio-maxillofacial surgery in the past, the following parameters were suggested when collecting data using a series of photographs:
  - The photographs should be in color.
  - The photographs should include lateral profiles from both sides, 45° photographs from both sides, and frontal photographs.
  - The photographs should be taken in the natural head position.

Despite these suggestions for clinical treatment planning, 2D photography has largely been superseded by 3D stereo-photogrammetry [3].

3. 3D scanning: 3D scanners can provide high resolution 3D data, including texture and color information, if required. Two types of 3D scanners can be used, i.e., facial scanners and intra-oral scanners. A disadvantage of using 3D scanners is that black-colored surfaces, such as hair, are not easily scanned and are often neglected [4]. Therefore, the 3D information can remain incomplete in certain situations.
4. CT/CBCT/MRI scanning: Tomographic data from traditional CT scans or cone-beam (CB) CT scans or MRI scans are now the preferred methods for clinical imaging. These techniques provide a series of planar images that can be easily concatenated to create a 3D object from digital data. One limitation of CT and CBCT imaging is the exposure to ionizing radiation that the patient must go through for the clinician to obtain relevant information. In this regard, CBCT scans are preferred over traditional CT scans due to their lower levels of radiation exposure [5,6]. However, although CBCT scanning is preferable, it can be less precise compared to traditional CT scanning on account of image clarity. In contrast, MRI scans do not deploy ionizing radiation. In some instances, particularly those where soft tissues are the primary concern [7], MRI scanning might be preferred; however, in some cases, it is difficult for a patient to hold steady for the duration of the scan. Nevertheless, some recommendations for CT/CBCT/MRI scanning for VSP are as follows:
  - Subject position: The patient should be standing or sitting in the natural head position, with the facial expression in repose. Currently, there are no guidelines on the stage of respiration [8], but chin rests and mouthpieces that affect the upper airway are best avoided for the sake of consistency.
  - Setup: The field of view should extend at least 10 mm beyond the outermost tissues in order to avoid distortions of any significant structures. The soft tissues especially should not be altered due to equipment fixtures and/or attachments, such as head stabilizers.
  - Resolution: If patient-specific tooling is required, a maximum resolution of 0.3 mm in all axes is required; otherwise, a maximum resolution of 0.5 mm can be used.

### 2.2. Segmentation and Visualization of the Virtual Model

**Figure 2:**
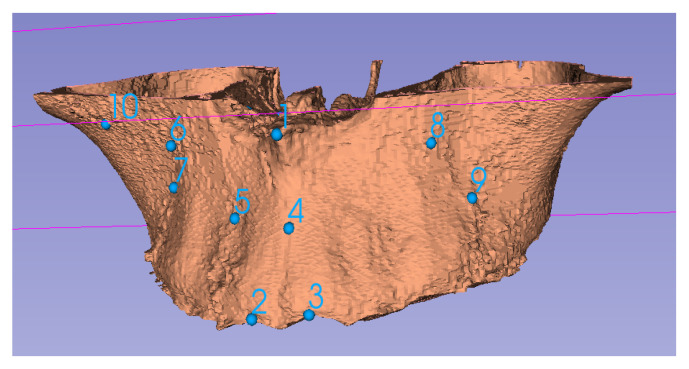
Segmentation of the midface with special attention on the maxilla and contiguous parts of the palatine bone, showing some homologous landmarks (1–10).

Anatomical features in a CBCT scan can be visualized using segmentation (surface rendering). For this process, specific thresholds in Hounsfield/grayscale units can be used to create differentiated surfaces. Using edge detection algorithms, various organs, such as the upper jaw (Figure 2), can be better visualized [9]. Segmentation can therefore provide different anatomical features, which can be used for VSP. Segmentation can also use Hounsfield unit information associated with each voxel to assign a specific color and transparency. Because of this facility, segmentation is useful for visualization but cannot be readily used for alignment; thus, homologous landmarks must also be identified (Figure 2). Sometimes, either due to noise or low resolution of a CBCT scan, various anatomical features cannot be accurately separated by simply using thresholds. In these cases, manual or semi-automated methods are utilized.

### 2.3. Alignment and Integration

After the data are acquired, they are most often accessed in the format of 3D point clouds, which have additional information associated with them. For example, 3D facial scans can include eye, skin and lip color as well as texture information. Conversely, CBCT scans can have density information. Regardless, these sources of information should be aligned properly to have all the data readily available for VSP. Many algorithms exist for the rigid alignment of point clouds. The most commonly used algorithm for point cloud alignment is the Iterative Closest Point (ICP) technique. During ICP, the algorithm computes the optimum translation and rotation of the objects repeatedly, to align a source point cloud to a target point cloud till the fit is no longer improved. During this procedure, the room mean square (RMS) distance of every point in the source object closest to its point in the target object is calculated. The total RMS distance for the entire point cloud is iteratively reduced by optimizing translation and rotation. These ICP techniques, such as Procrustes superimposition, have been thoroughly tested for medical applications [10] and have been found to be useful in this regard [11].

### 2.4. Virtual Surgery Planning

**Figure 3:**
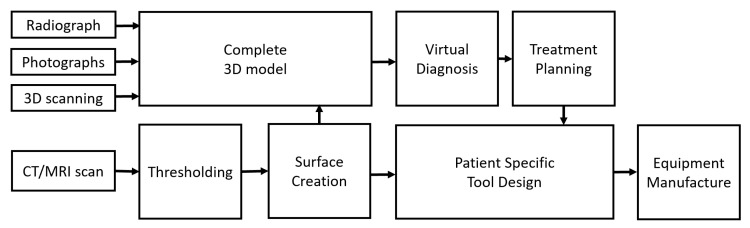
Detailed VSP procedure.

**Figure 4:**
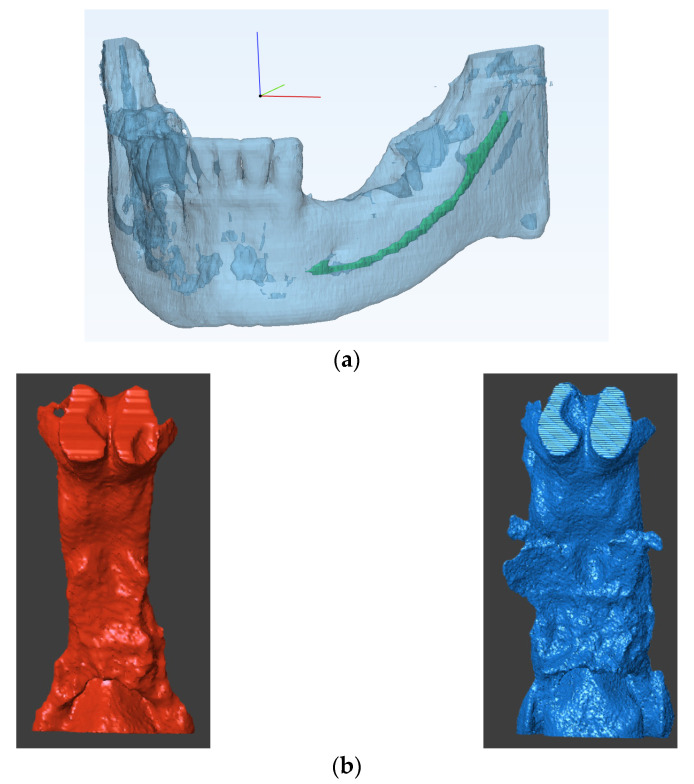
(**a**) Visualization of nerve location in the mandible for tool design. (**b**) 3D printing of upper airway before (red) and after treatment (blue).

After going through the steps noted above, the next stage is the actual 3D VSP. This stage requires training in the use of the various planning and visualization tools (Figure 3) encoded by the software being utilized. Therefore, a bioengineer or clinical technician is often required to assist the medical practitioner. The major steps in VSP are as follows:

1. Virtual diagnosis: The first and most important step is proper diagnosis. Various analytical tools are often available for the measurement of 3D data. These features can be used to quantify the defect, deficiency or dysmorphology, which might not be possible using traditional surgical planning methods. Note that Euclidean measurements integrate size information, which may mask subtle shape changes associated with clinical behavior.
2. Treatment planning: Once the differential diagnoses have been excluded and a working diagnosis has been achieved and quantified, optimum plan parameters can be calculated. Craniofacial surgical plan parameters generally include, but are not limited to, an osteotomy location and angle. A virtual osteotomy can thus be simulated, and the final alignment accuracy can be checked. While this approach is summarized in Figure 3**,** the virtual osteotomy in this example is crucial yet sensitive to the expertise of the technician, making it the weakest point of the VSP as it lacks automation and relies on human decision making. Here, predictive modeling comes into play. According to the spatial matrix hypothesis [12], historically speaking, there is a tacit assertion that the craniofacial complex consists of a series of structural components of genetically predetermined form. Generally, surgically induced changes of these biologically active structures are simply perceived as differential movements. These concepts do not permit the dynamic, biologic behavior of the craniofacial structures to be taken into account, which constantly regress to homeostasis and perhaps lead to relapse in some instances. Conversely, using a cohort of cases that have had the same surgical intervention, it should be possible to compute the mean, underlying 3D transformation for a sample of cases, using techniques derived from mathematical modeling, including geometric morphometrics [13]. If this transformation can then be applied to a naïve subject, a predictive model can be achieved, assuming the new subject behaves in the same way that the sample did on average. This novel data-driven predictive modeling is unlike the animations that are used for arbitrary ‘morphing’ in some orthodontic software. Therefore, the use of mathematical modeling on 3D digital data provides a promising avenue of research in terms of VSP.
3. Patient specific tool design: Using this approach, patient specific parameters, such as bone thickness, nerve location etc., can be visualized (Figure 4a). If required, patient specific tools and surgery guides can be designed using surgery plan parameters, such as osteotomy location and screw placement.

### 2.5. Manufacturing

Three-dimensional models can also be printed from digital data, such as the ones illustrated in Figure 4b to inspect post-treatment outcomes. This process is often required if patient-specific tools need to be produced in order to perform the operative procedure. Patient-specific tooling can include either surgical tools, such as osteotomy guides and screws, or patient models that can be used as templates, such as mesh plate bending, or both. Either way, patient-specific tooling is manufactured using 3D printing directly or indirectly. Based on the type of application, an optimum biocompatible material is selected. A manufacturing technician usually plans the 3D printing parameters for every component to be manufactured. The printing parameters are selected to improve model strength, reduce the overall weight and manufacturing time. In addition, post-printing processes, such as chemical etching for improved osseointegration, can also be deployed. Therefore, communication between the surgical planner and the manufacturer is crucial, and a checklist for proper communication can be proposed such that 3D models can be printed, such as the ones illustrated in Figure 4.

## 3. Prediction Based Virtual Surgery Planning

A disadvantage of VSP’s current workflow is the significant learning curve associated with the process. There are many potential sources of error in the entire procedure for successful surgical planning, such as image processing, segmentation, alignment, selection and use of visualizing algorithms for virtual diagnosis. The workflow must be designed to make the overall procedure immune to such sources of errors. Traditional surgery planning procedures are limited by the fact that they mostly utilize population specific parameters. Conversely, current VSP methods are limited by the fact that they mostly use patient-specific parameters. The use of population-specific parameters in addition to patient-specific parameters can enhance the planning process and improve the results. Despite that, VSP is not preferable in cases of fast changing patient anatomies, such as various tumors. Current VSP methods assume that the patient’s anatomy does not change significantly between the time of planning and the time of surgery. This lead time can sometimes be significant in more complicated cases, since both the planning time and manufacturing time might be increased. Yet another limitation of VSP is that there is no proper feedback mechanism for continuous improvement of the planning process. The surgeons must rely on their experience and available literature to improve the planning.

**Figure 5:**
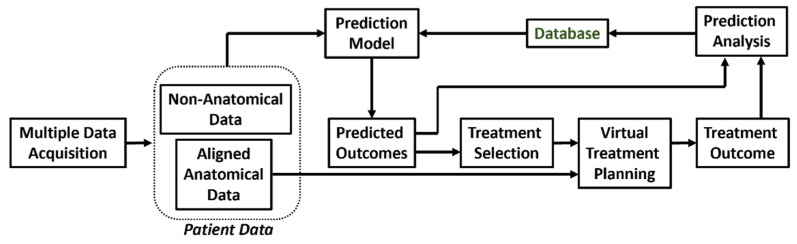
Schematic diagram of prediction based VSP.

The above limitations of VSP in its current form are being realized and new approaches to prediction-based surgery planning are beginning to emerge. The schematic for prediction-based VSP is illustrated in Figure 5. The key difference of this scheme is that it has a closed loop architecture instead of the open loop architecture used for traditional VSP. Another major difference is that the decision for treatment selection, which is traditionally a part of the virtual diagnosis, and depends highly on the medical practitioner, is now assisted by the results of a predicted output model. The process starts with the acquisition of patient data from multiple sources. Similar to traditional VSP, these data are aligned to obtain a full scope of patient anatomy. However, in addition to anatomical information, non-anatomical data specific to the patient is also collected. The complete data (anatomical and non-anatomical) is then used by the prediction model to forecast the outcome of a selected treatment plan. Using this approach, patient-specific chances of success and the risks of various treatment options can be compared, by using the prediction model outcomes of alternative treatment plans. For fast-changing anatomies, prediction models using Bayesian statistics and Monte Carlo simulations can be used to accommodate changes in the anatomy associated with the time delay before surgery. In these cases, VSP and the development and manufacturing of patient-specific tools is performed, as performed traditionally. The final treatment outcome is then recorded and analyzed against the predicted outcomes of the selected treatment prediction model. Deviations of the actual outcome from the predicted model are calculated and recorded in a database. This database is then used for continuous improvement of the prediction model (Figure 5).

This novel methodology helps the clinical planner in selecting the most appropriate treatment method and parameters for a particular patient. In addition, predictive models help the patient by providing a range of realistic outputs of the various surgical procedures. This approach is more apt because the predictive models use real life data, which includes various sources of error, e.g., in planning, during surgical implementation and errors due to patient response. Note that ‘errors’ here includes and refers to statistical variation associated with distribution of normative data, unlike arbitrary pixel movements on a computer screen. In addition, while some predictive techniques try to forecast non-definitive parameters, such as possibility of success, risk associated with a particular treatment, survival rate, possibility of a disease in the future, etc., in the current work, only shape and size prediction methods are discussed. Various efforts have been made to predict the anatomical outcome of a specific surgical intervention. These predictive methodologies can be categorized as:

1. Model-based prediction methods;
2. Data-driven predictive modeling;
3. Hybrid prediction methods.

### 3.1. Model-Based Prediction

Model-based prediction methods forecast the shape change, with or without size scaling, by using mathematical modeling predicated on physical sciences to solve for optimum prediction. These allometric, model-based methods usually discretize the anatomy into elements. The changes are predicted in every element individually and then combined to give the overall prediction. The major advantages of using model-based prediction are:

1. A large database is not required to develop an accurate model. Therefore, the methods are directly applicable for procedures in which data are not easily available.
2. Properties quantified and/or measured in ideal conditions (such as tissue density, elasticity, stiffness, etc.) can be directly used.
3. Both population and individual parameters are taken into account for prediction. The underlying mathematical model considers population-specific parameters, and the discretized anatomy considers the individual parameters.

The limitations of model-based prediction methods are:

1. Solving non-linear models is time-consuming.
2. Developing and using model-based methods is difficult.
3. The method cannot be used in conditions where the parameters of the underlying mathematical model are unknown.
4. Changes unaccounted for in the mathematical model cannot be predicted.

Some commonly used model-based prediction methods are:

- Finite element method (FEM): FEM is an old but reliable method. This technique finds extensive use in bioengineering since FEM can be used to solve any set of partial differential equations on the nodes of the discretized model. For observing the change in shape, structural equilibrium equations are used (Equation (1)). Here, σ and τ are the normal and shear stresses in an element under consideration, F is the force, and x, y, z are the directions in Cartesian coordinate systems.
  (1) [σxxτxyτxzτyxσyyτyzτyzτzyσzz][∂∂x∂∂y∂∂z]+[FxFyFz]=0 
  Tissues can be modeled considering linearity and elasticity models [14]. Therefore, for realizing the relation between stress and strain, Hook’s law is used, and both linear and non-linear behavior can easily be modeled using FEM. Since FEM can simulate details of the non-linear behavior of tissue structures, it has been used for prediction of lip [15,16] and facial soft tissue changes [17].
- Probabilistic FEM [18,19]: Often, the exact material properties are not known. However, the material properties vary within a known range. In such a situation, the use of probabilistic FEM is preferred. In this method, the equations used for simple FEM are solved over a defined interval instead of a defined constant value. This approach is often regarded as a subclass of FEM.
- Mass spring model (MSM): This model consists of discrete mass nodes distributed throughout an object and interconnected via a network of springs and dampers. Therefore, the solutions of MSM are considered linear in nature. MSM is suitable for modeling objects with complex material properties, such as non-linearity and viscoelasticity and has been utilized extensity in the literature [20,21,22,23], but the challenge with MSM is always the correct approximation of spring constants.
- Mass tensor model (MTM) [24,25]: This model tries to combine the advantages of FEA and MSM and provides quasi-linear solutions. For example, it can be used to simulate soft tissue displacement after bone deformation. It uses tetrahedral or triangular elements to approximate the shape, making it fast, but also placing an upper limit on the complexity that MTM can handle [26]. Table 1 compares the advantages and limitations of commonly used model-based prediction methods.

### 3.2. Data-Based Prediction

Data-based prediction methods do not require a mathematical model to forecast the outcome of a surgical procedure. The prediction uses only the constraints of the data from previous instances. The predictive model can be based on statistical methods or using advanced, artificial intelligence (AI) techniques. The advantages of using data-based predictive models are:

1. Applicable in most cases and do not require a specific mathematical model for defined conditions.
2. Easier to learn, develop and implement as compared to model-based methods.
3. Can provide complex results relatively rapidly.
4. When using machine-learning algorithms, the accuracy improves with every prediction.

The major limitations of using data-based algorithms are:

1. They require a large database, which might not be readily available.
2. The precision of even highly accurate models is limited by their training database. Thus, a patient-type or procedure-type that is not extensively referenced in the training database cannot be assured to produce an accurate prediction.

Other techniques use tools from statistical mathematics to find a predicted model. Commonly used statistical methods for creating a predictive model are as follows.

- Principal components analysis (PCA): PCA finds its origins as a model reduction technique. PCA provides new components that are a linear combination of the original components of data. The new components are formed to capture most of the variation in the data, using the least number of components. PCA is, therefore, a useful technique for data analysis and outlier detection. Using this approach, the underlying behavior of the data can be identified and described in statistical shape space as a series of eigenvalues. Thereafter, eigenvectors can be applied to a new dataset to visualize possible outcomes. Thus, PCA is particularly useful as a data pre-processing technique in predictive modeling and is the most commonly used data-based method [27].
- Euclidian distance matrix analysis (EDMA): Since some are of the opinion that principal components analysis (PCA) methods, based on superimposition, can lead to an incorrect estimation of form and variance–covariance structures, EDMA has been advocated as an alternative [28]. Euclidean distance is one of the most used metrics, and EDMA is a space ratio method, providing a coordinate-free approach to the analysis of form using landmark data, which may provide another approach to data-driven predictive modeling.
- Principal Shape Analysis (PSA): A common limitation in PCA is the difficulty in relating the components modes with the intuitive shape descriptions used by clinical practitioners. Aguirre et al. [29] proposed the use of PSA to counter this problem. PSA models covariance between variables rather than the total covariance in the data. Hence, PSA provides better interpretation by proving the components that can account for the variance in the entire dataset.

The statistical prediction models map the input anatomy and other treatment variables to an output anatomy. The input–output relationship is then approximated using regression. Many types of regression such as principal least squares (PSL), gradient boost and random forest (RF) can be used. The selection of the regression model depends on the accuracy required by the given dataset. Machine learning (ML) methods can also be used instead of regression; however, AI methods have been proven to outperform simple regression, by accommodating many other parameters. Nevertheless, these AI methods, especially those using supervised learning algorithms, require large datasets and proper labeling of data. Recently, machine learning was used for both diagnosis and prediction of facial surgery outcomes [30]. Such ability to perform multiple tasks in a single step is a crucial advantage in the use of machine learning. Table 2 summarizes the advantages and limitations of these various prediction methods.

### 3.3. Hybrid Prediction

As discussed previously, both model-based and data-based methods have their inherent advantages and disadvantages. In order to overcome these flaws, a combination of model-based and data-based techniques can be used. Previously, Zolfagharnasab et al. [31] created a hybrid prediction model to forecast breast shape after breast correction surgery. The slow speed of purely model-based prediction was overcome by training a data-based model to mimic the results from a model-based prediction method. In addition, the unavailability of data for purely data-based modeling was overcome by accurate simulation, using a model-based prediction method. Liao and Köttig [32] discussed possible hybrid combinations for failure predictions in systems engineering, where hybrid models are extensively used. For surgery prediction, similar hybrid models can be also used, in the following possible settings.

- Model + Model: Two or more models can be used to make predictions. For example, combination of a biochemical model for wound healing with a biomechanical tissue deformation model was used for surgical prediction by Vavourakis et al. [33].
- Data + Data: Two data-driven models can also be used for improving the overall accuracy of the prediction. For example, different regions of the same anatomy can be analyzed separately to create separate prediction models. The results from all the models can be combined to have an overall prediction. Bayesian-based, patient-specific growth models for anatomical changes associated with fast changing anatomies have shown promising results.
- Model + Data: This is probably the most powerful hybrid model with many possible applications. Model-based methods and data-based methods can be combined to perform the following tasks:
  1. A data-driven method can be trained to mimic the results and behavior of the model-based method. The faster data-driven model can then replace the slower mode-based method [34,35].
  2. A model-based method can be used to simulate and fill in the gaps in the database used for data-based prediction method. Jiang et al. [36] and Vavourakis et al. [33] utilized such techniques for predicting changes in abdominal aortic aneurysms and breast tissue deformation, respectively.
  3. Data-driven techniques can be used to create population-specific parameters required by model-based methods [37].
  4. A data-driven method can be used to predict the boundary conditions required for model-based method. The model-based method can then make accurate predictions [38].
  5. Both data-based methods and model-based methods can make individual predictions, which can be fused to provide a highly accurate, final prediction.

## 4. Selection of Prediction Method

**Figure 6:**
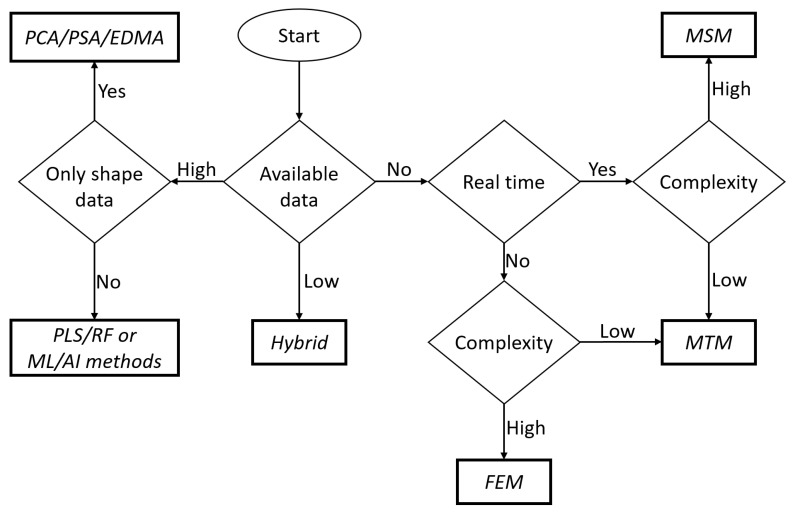
Algorithm for selection of prediction method.

For the proposed prediction-based workflow, selection of the most appropriate prediction method is necessary. There are no specific guidelines for this selection; however, a scheme for this decision making is shown in Figure 6. For example, if data from previous procedures are not available, then a model-based method is required. For low amounts of data, using a single data-based method can be unreliable; therefore, a hybrid method should be used either by combining a data-based method with a model-based method or by integration of multiple data-based models created from the available data. If large amounts of data are available, data-based methods should be preferred over model-based methods.

When model-based methods need to be developed, both the model complexity and requirement of real-time prediction should be considered. If the procedure requires real-time prediction (e.g., for robotic surgery), then it is unnecessary to create different prediction models because the final results will be governed by the real time prediction method utilized. In such a situation, the solving speed should be prioritized over accuracy such that the procedure does not malfunction mid-procedure due to a slow solving speed. Therefore, for highly complex modeling, MSM is used because of its simplicity. For low complexity prediction models, MTM is more beneficial, as it can provide better accuracy without depleting the system resources.

If the model-based method does not need to be solved in real time, then accuracy can be given priority over solving time. Therefore, for complex models the highly accurate FEM should be preferred. For low complexity, if the accuracy of MTM is comparable to that of FEM, MTM should be used to save VSP time. In addition, when considering data-based models, the final model selection depends on the type of data available. If only pre- and post-operative shape information is available, it is suggested to use statistical shape analyses, such as PCA and PSA. Furthermore, if the data contain other treatment- and/or patient-specific information, then a more complex mapping model, such as machine learning or artificial intelligence, can be used. These approaches are summarized in Figure 6.

## 5. Prediction Analysis

**Figure 7:**
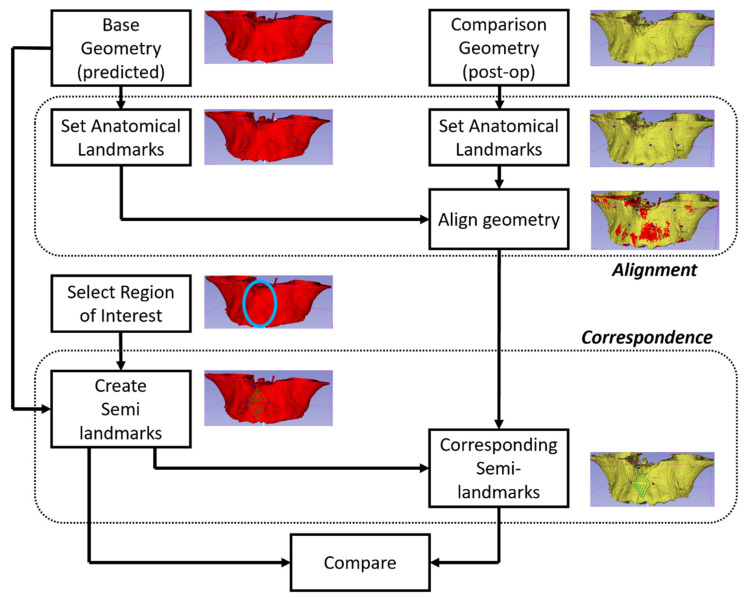
Prediction analysis procedure.

Analyzing the accuracy of a predicted model’s outcome is important to avoid making erroneous assumptions regarding clinical tissue behavior. The accuracy and precision of predicted models need continuous improvement over time. Therefore, proper and ongoing analyses of predictive modeling techniques are necessary. A predicted model can be analyzed by comparing the predicted outcome in terms of shape, size and positional information to the actual outputs. Therefore, a visual 3D model of the patient’s post-treatment configuration is necessary. The prediction analysis process can be understood using Figure 7. To compare the predicted and actual post-treatment configurations, both objects must be aligned, using homologous landmarks. This process brings both objects into comparable special coordinates. The region of interest is then selected, and semi-landmarks [39] are populated in both objects. Semi-landmark are points that correspond to each other at anatomical locations. They differ from homologous landmarks since they are calculated in specific, but not in generic, morphologies. Thus, corresponding semi-landmarks are compared.

Assessment of precision of any predictive method may entail some variations in steps, as follows.

### 5.1. Landmark Selection

The anatomical object is represented mathematically, using a point-cloud. The points in the point-cloud are joined together using edges. Subsequently, the edges are collected to represent a surface. Landmarks need to be placed on the surface to analyze it and also to compare it to other surfaces. These landmarks can be derived directly from the homologous landmarks in the point-cloud or created, using mathematical functions. Three types of landmarks are used:

1. Anatomical landmarks: These are called homologous landmarks, which are agreed by experts to represent various anatomical features.
2. Mathematical landmarks: These are the points on the surface located according to a specific geometric property, such as maxima or minima of high curvature.
3. Pseudo-landmarks: These are also called semi-landmarks and are points that are constructed using other types of landmarks and mathematical logic.

**Figure 8:**
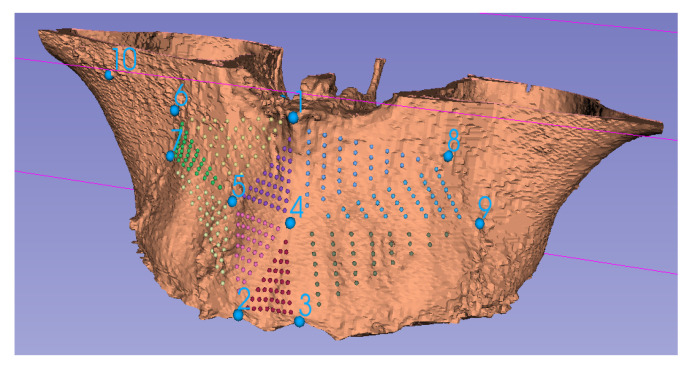
Using CBCT data, the maxillary complex has been rendered in 3D and the semi-landmarks (colored points) have been computed using the ten homologous landmarks (1–10).

For assessment of accuracy of a predictive model, a 3D point-cloud representing the size and shape of the anatomic structure of the predictive model is accessed as an initial input. The prediction algorithm transforms this point-cloud to form a data-driven predictive model. This predictive model is then compared to the final shape, size and positional data post-treatment to test the goodness of fit and test how closely the predictive model matches the actual outcome. Therefore, the comparison is centered on point-clouds based on the original anatomical, homologous landmarks after alignment (say, using Procrustes superimposition), calculated landmarks (or semi-landmarks, Figure 8) or those established through dense correspondence [4]. To assess the level of precision and accuracy, differences can then be quantified using, say, RMS, EDMA, or absolute Euclidian distance.

### 5.2. Automated Landmark Detection

The prediction analysis process is highly dependent on landmarks and requires their placement at every stage. Traditionally, cephalometric analysis was performed on radiographs, which are 2D projections of craniofacial anatomy. For this analysis, manual landmark placement is preferred for accuracy and reliability even today. However, analysis of 3D images has some challenges. First, currently there is no consensus for 3D landmarks; thus, for 3D analyses, the equivalent counterparts of recognized 2D landmarks are selected [40]. Second, accurate manual landmark placement on 3D objects is a time-consuming process and requires high levels of experience. For example, the time for manual placement was observed to be up to 14 min. for a single case [41]. Hence, to create a large database required for prediction, automated landmark detection might be advisable. Automated landmark placement methods can be categorized into three types [40], as follows:

a. Knowledge based [42]: These methods use mathematical definitions such as curvature, maxima, minima, etc. to locate the landmark on an anatomical contour of the 3D object or surface.
b. Atlas based [43]: In these methods, before automatic placement begins, a reference atlas is created using a manual landmarking process. The reference atlas is assumed to be accurate and inclusive. For automatic placement, the subject under consideration is matched with the reference elements in the atlas. The closest matching reference is used to transfer the landmarks from the selected reference to the subject.
c. Learning based: These methods rely on learning algorithms and available datasets to automatically locate landmarks. The learning methods employed can either be statistical or based entirely on machine learning.
  - Statistical learning methods correlate the object with either deformation modes called active shape models [44] or a graphical representation called elastic bunch graph matching [45] extracted from the available dataset.
  - Machine learning based methods either create a categorization method called the random forest method [46] or directly approximate the landmark location using deep-learning architecture [47]. The methods of automatic landmark placement are compared in Table 3.

Other points to consider when selecting an automated landmark placement method are the robustness of the technique to image noise, anatomical variations, accuracy of the final placement and time consumed.

### 5.3. Alignment

After landmarks placement, the anatomical surfaces are aligned to permit comparisons. Therefore, it is necessary to assure accuracy and precision in the alignment of the structures under consideration. Alignment can be done for quantifying the changes for the same patient post-surgery or for comparing the post-surgery changes among different patients.

- For analysis of morphology of the same patient, usually the non-morphing configurations are used as the reference. Homologous landmarks are placed on locations that do not change due to the treatment. The pre-treatment and post-treatment objects are then translated and rotated iteratively for optimal alignment. Note that scaling is not performed in this technique unless natural growth needs to be accommodated.
- When analyzing and comparing configurations from different patients or if an unaffected reference is are not available, generalized Procrustes analysis (GPA) is used to align homologous landmarks as close as possible. The Procrustes mean is then calculated. The configurations are then aligned to the mean using thin plate spline (TPS), explicitly minimizing the bending energy [39].

### 5.4. Region of Interest (ROI)

After alignment is established, the region of interest for analysis is selected. Statistical analysis is then required to create a population-level predictive model. In the literature, the following ROIs are usually considered:

- Total anatomy: The entire geometry for which point correspondence is available can be considered. The advantage of such ROIs is that it is easier to implement. The disadvantage is that the change due to treatment is localized, therefore, tracking the actual change becomes difficult as the numeric values are dominated by unchanged parts of the anatomy. When the entire anatomy is analyzed, it is also important to adjust for outliers. Two methods have been employed in the literature for handling outliers. First, the absolute distance between the points from the two objects is ordered in decreasing magnitude. Instead of using all the points, only a certain lower percentile is used, traditionally the 90th percentile. An alternate method is to use a fixed tolerance where points that lie outside the tolerance are neglected from analysis; traditionally, a 2 mm tolerance is accepted.
- Local area: In this case, instead of using the entire geometry, only a local area is selected. The advantage is that changes are easier to track. The disadvantage is that an additional step (manual, automatic or semi-automatic) is often required to segment the local ROI. For analysis of a local ROI, semi-landmarks can preferably be used.
- Local curve: Sometimes instead of using a local ROI, a local curve is considered. This can be useful in certain applications such as prediction of the facial midline, shape of the spine, etc. For generation of curves, mathematical landmarks are used.

### 5.5. Correspondence

Implementing correspondence between point clouds is usually the most time-consuming step in the analysis. For correspondence between the same ROI from different sources (either from the same or different patient) the ICP algorithm can be used. One of the point clouds is designated as the source or target and the other is designated as the reference. The reference point cloud is then iteratively rotated such that both point clouds align optimally. ICP has been found to be highly accurate for medical applications [48].

### 5.6. Difference Quantification

Finally, after establishing correspondence, the difference between the predicted and actual outcome can be quantified. The difference can be considered for only size or both shape and size. For difference in size, distances between corresponding points in the ROI are calculated. The point-to-point distances can be Euclidian distance, RMS distance, or absolute Euclidian distance. Euclidian distance gives the signed difference between the corresponding points; therefore, an increase or decrease in size can be differentiated. However, the signed difference is not suitable for calculation of parameters such as mean or median change. For calculation of such parameters, the unsigned values of change are required. A common unsigned difference is the RMS change. The limitation of using RMS change is that due to the square operation, an excessive emphasis is given to large differences in points. Therefore, the absolute Euclidian distance is often used whenever an unsigned distance is required.

For shape and size quantification, Finite element analysis (FEA) is commonly used. This should not be confused with the FEM used for prediction. Both approaches share a similar name because both can use an approximate, simple configuration to analyze a complicated anatomical object. Finite elements used for shape and size change quantification usually consist of polygons created using anatomical landmarks. The lengths of the sides of the polygons and the associated angles can be used to observe the change in both size, shape and directionality simultaneously. Concentrated data, where a large number of homologous landmarks are clustered compared to distant ones, is a potential disadvantage of FEA. Therefore, another possible method for shape and size quantification is to calculate the cartesian components of the point-to-point differences.

## 6. Discussion

The need for robust surgical outcome prediction is increasing since surgical options are coming under further scrutiny, while the plethora of techniques is proliferating. Therefore, it is becoming more essential to select the most appropriate surgical procedure for a particular patient. In this regard, Gracia-Abuter et al. [49] are of the view that 3D image acquisition using CBCT scans permits the construction of dynamic and interactive 3D visual models, which in turn allow accurate or predictable VSP and efficient surgical procedures for patients with complex craniofacial issues.

Borba et al. [50] studied mean discrepancies for measured parameters between predicted models obtained with VSP and those obtained clinically for orthognathic cases. They reported values of <2.0 mm ± 2.0 (standard deviation) for most maxillo-mandibular landmarks, after manual superimposition. In addition, the accuracy of the surgical movements predicted were not significantly different for most landmarks, regardless of gender and type of deformity. In a small study, Quereshy et al. [51] also concluded that VSP is useful for orthognathic surgery since it can yield favorable and accurate outcomes. Later, in a sample of over 60 patients, Kwon et al. [52] investigated discrepancies between actual and planned movements in segmental maxillary osteotomies. On average, there was a 0.96 mm ± 0.69 discrepancy transversely, 1.23 mm ± 0.83 vertically, and 1.16 mm ± 0.80 antero-posteriorly between clinical and VSP movements. Similarly, Qadry et al. [53] compared pre-operative, planned and post-operative results among various craniofacial treatment groups. Their results revealed that the differences between pre-operative and post-operative measures were statistically significant (*p* < 0.05), as expected, and the differences between pre-operative measures and the planned configuration were also statistically significant. However, differences between the planned configuration and the post-operative measures were non-significant (*p* > 0.05), suggesting that VSP using patient-specific splints and cutting guides designed for accurate transfer of placement might be reliable. However, these broad approaches do not aid in selecting which surgical technique best suits an individual patient.

In clinical practice, it is essential to localize the site and severity of the dysmorphology, if at all possible. Taking an empirical approach to this complex question, Tecuta-Busoi et al. [54] were able to identify diminution of the posterior cranial base in patients diagnosed with Turner syndrome. Here, precise localization of the anomaly as well as quantification of the deficiency was not fully achieved since 2D cephalometry was the analysis of choice. Nevertheless, these types of studies provide valuable baseline information in the development of 3D data-driven predictive modeling since historical data are a crucial starting point to test the robustness of any novel routines. Conversely, Park et al. [55] compared the efficacy of conventional surgical planning with VSP. They found a 41% to 62% reduction in time when deploying VSP compared to traditional methods, but there was no direct reduction in costs, which were attributed to indirect cost saving because of the reduced labor cost due to reduced time. These types of findings are useful since the time, effort and expense of VSP should not be onerous if widespread adoption is foreseen in the future. As existing manual technologies change to robotic approaches, the use of VSP may permit data integration into smart devices and hardware that includes wireless, miniaturized, implantable components, encapsulated by biocompatible materials, as wearable technologies that overcome analogue disadvantages of existing clinical standards. For example, in OSA, after VSP, customized bioresorbable airways stents could be produced by 3D printing [56] and further applications in orthognathic surgery and oncologic reconstruction are envisioned. While these approaches provide promising perspectives for manufacturing customized medical devices with accuracy, precision and intelligence, for clinical practice, it is concluded that VSP might become an essential tool in selecting the most appropriate surgical procedure for a particular patient.

## Figures and Tables

**Table 1:** Comparison of FEM, MSM and MTM model-based prediction methods.

Technique	Advantages	Limitations
Model based techniques	Does not need huge amounts of data Usually have a biomechanical or biochemical foundation	Difficult to create the model Different models might be required for different populations i.e., a single model cannot capture the entire population
Mass spring model	Simple architecture Low computation cost	No real biomechanical foundation Requires significant pre-computation Does not have full dynamic behavior
Mass tensor model	Is quasi-linear, thus is highly accurate for small deformations	Supports only tetrahedral elements; thus, model complexity is limited. Requires pre-computation
Finite element model	Accurate Strong biomechanical foundation Well-developed technique even for complex situations such as ruptures	High computational cost Difficult architecture

**Table 2:** Comparison of data-based prediction methods.

Method	Advantages	Limitations
Data based methods	Easy and quick implementation Low computation cost implementation All contribution factors are already captured in real data	Requires huge amounts of data Population specific models need to be created No clear biomechanical foundation
Statistical methods	Strong mathematical foundation	Cannot capture highly non-linear relations Usually based on model reduction techniques such as PCA; thus, some data are lost
ML/AI methods	Can handle complex non-linearities A single model can model a variety of population	Accuracy is algorithm specific

**Table 3:** Comparison of automated landmark placement methods.

Method	Advantages	Limitations
Knowledge-based	Conforms to agreed mathematical and anatomical definitions Does not require high computational power	Contour detection is prone to errors Landmarks can be difficult to locate on curved surfaces Not robust for severe deformities Sensitive to image noise
Atlas-based	Easy to customize and improve Requires small database	Atlas must be accurate and inclusive (age, sex, ethnicity, physical features) Performance depends on point registration technique used
Learning-based	Less sensitive to image noise Can accommodate large variations in coordinates	Requires a large database Not all techniques are applicable in 3D space Training parameters need to be determined empirically
